# Crystal structure of *S*,*N*-dibenzyl-d-penicillamine monohydrate

**DOI:** 10.1107/S1600536814023459

**Published:** 2014-10-29

**Authors:** Nobuto Yoshinari, Takumi Konno

**Affiliations:** a Department of Chemistry, Graduate School of Science, Osaka University, Toyonaka, Osaka 560-0043, Japan; b CREST, Japan Science and Technology Agency, Toyonaka, Osaka 560-0043, Japan

**Keywords:** crystal structure, hydrogen bonds, penicillamine

## Abstract

In the asymmetric unit of the title compound, C_19_H_23_NO_2_S·H_2_O, there are two independent organic mol­ecules and two water mol­ecules. Both organic mol­ecules exist as the zwitterionic form. The dihedral angles between the planes of the rings in the organic mol­ecules are 86.84 (10) and 88.77 (11)°. An intramolecular N—H⋯S hydrogen bond occurs. In the crystal, organic and water mol­ecules are linked by N—H⋯O and O—H⋯O hydrogen bonds, generating a tape structure running along the *b-*axis direction.

## Related literature   

For the synthesis of the title compound, see: Crooks (1949 ▶). For the coordination behavior of d-penicillamine and its derivatives, see: Igashira-Kamiyama & Konno (2011 ▶); Oji *et al.* (2014 ▶).
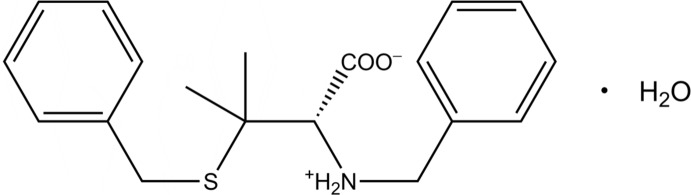


## Experimental   

### Crystal data  

C_19_H_23_NO_2_S·H_2_O
*M* *_r_* = 347.46
Monoclinic, 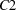

*a* = 19.930 (2) Å
*b* = 6.2500 (7) Å
*c* = 30.645 (4) Å
β = 98.715 (7)°
*V* = 3773.2 (8) Å^3^
*Z* = 8
Mo *K*α radiation
μ = 0.19 mm^−1^
*T* = 200 K
0.15 × 0.10 × 0.03 mm

### Data collection  

Rigaku R-AXIS RAPID diffractometer
Absorption correction: multi-scan (*ABSCOR*; Higashi, 1995 ▶) *T*_min_ = 0.778, *T*_max_ = 0.994
15147 measured reflections
7694 independent reflections
7006 reflections with *I* > 2σ(*I*)
*R*_int_ = 0.027

### Refinement  

*R*[*F* ^2^ > 2σ(*F* ^2^)] = 0.041
*wR*(*F* ^2^) = 0.093
*S* = 1.06
7694 reflections
461 parameters
1 restraint
H atoms treated by a mixture of independent and constrained refinement
Δρ_max_ = 0.44 e Å^−3^
Δρ_min_ = −0.25 e Å^−3^
Absolute structure: Flack *x* determined using 2566 quotients [(*I* ^+^)−(*I* ^−^)]/[(*I* ^+^)+(*I* ^−^)] (Parsons *et al.*, 2013 ▶)
Absolute structure parameter: 0.03 (2)

### 

Data collection: *PROCESS-AUTO* (Rigaku, 2000 ▶); cell refinement: *PROCESS-AUTO* (Rigaku, 2000 ▶); data reduction: *PROCESS-AUTO* (Rigaku, 2000 ▶); program(s) used to solve structure: *SHELXS2013* (Sheldrick, 2008 ▶); program(s) used to refine structure: *SHELXL2013* (Sheldrick, 2008 ▶); molecular graphics: *Yadokari-XG* (Kabuto *et al.*, 2009 ▶); software used to prepare material for publication: *Yadokari-XG* (Kabuto *et al.*, 2009 ▶).

## Supplementary Material

Crystal structure: contains datablock(s) I, New_Global_Publ_Block. DOI: 10.1107/S1600536814023459/is5378sup1.cif

Structure factors: contains datablock(s) I. DOI: 10.1107/S1600536814023459/is5378Isup2.hkl

**Fig. 1.**
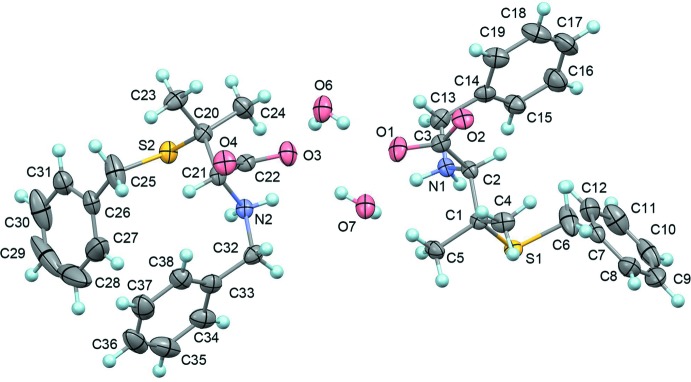
The asymmetric unit of the title compound with the atom numbering scheme. Displacement ellipsoids are drawn at the 50% probability level.

**Fig. 2.**
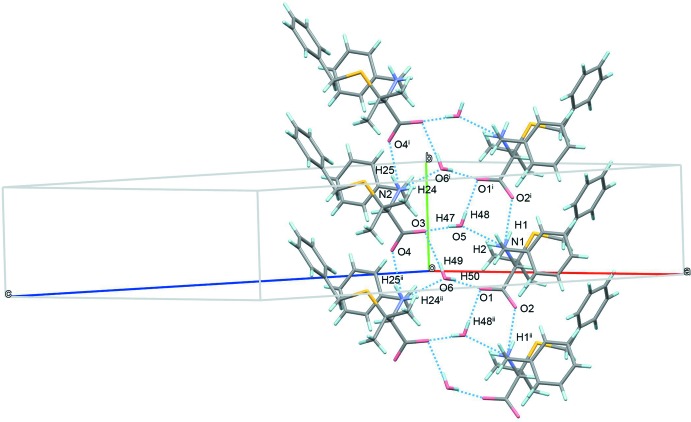
A view of the tape structure running along the b axis in (I). Blue dashed lines indicate hydrogen bonds. [Symmetry codes: (i) x, y + 1, z; (ii) x, y–1, z.]

. DOI: 10.1107/S1600536814023459/is5378fig1.tif
The asymmetric unit of the title compound with the atom numbering scheme. Displacement ellipsoids are drawn at the 50% probability level.

b x y z x y z . DOI: 10.1107/S1600536814023459/is5378fig2.tif
A view of the tape structure running along the *b* axis in (I). Blue dashed lines indicate hydrogen bonds. [Symmetry codes: (i) *x*, *y* + 1, *z*; (ii) *x*, *y*–1, *z*.]

CCDC reference: 1030824

Additional supporting information:  crystallographic information; 3D view; checkCIF report

## Figures and Tables

**Table 1:** Hydrogen-bond geometry (, )

*D*H*A*	*D*H	H*A*	*D* *A*	*D*H*A*
N1H1S1	0.89(3)	2.67(3)	3.107(2)	112(2)
N1H1O2^i^	0.89(3)	2.02(3)	2.835(3)	152(2)
N1H2O5	0.94(3)	1.89(3)	2.761(3)	154(3)
N2H24O6^i^	0.88(3)	1.96(3)	2.774(3)	155(3)
N2H25S2	0.87(3)	2.67(3)	3.112(2)	113(2)
N2H25O4^i^	0.87(3)	2.06(3)	2.851(3)	151(3)
O5H47O3	0.81(3)	1.97(3)	2.781(3)	172(4)
O5H48O1^i^	0.80(4)	2.03(4)	2.824(3)	173(3)
O6H49O3	0.81(4)	2.13(4)	2.927(3)	171(3)
O6H50O1	0.81(3)	2.03(4)	2.825(3)	169(4)

Symmetry code: (i) 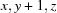.

## supplementary crystallographic information

### S1. Comment

As a part of our ongoing studies on the synthesis and structures of the transition metal complexes with *D*-penicillamine (Igashira-Kamiyama & Konno, 2011), we recently tried to prepare metal complexes with D-penicillamine derivatives (Oji *et al.*, 2014). We report herein the structure of the title compound (I), which was accidentally obtained in the course of the preparation of *S*-benzyl-*D*-penicillamine from benzyl chloride and *D*-penicillamine. This compound (I) has been synthesized but has not been structurally characterized (Crooks, 1949).

### S2. Experimental

A mixture containing *D*-penicillamine, sodium hydroxide, and benzyl chloride in a 1:3:3 molar ratio in water was stirred in an ice bath overnight. The resulting colorless solution was neutralized by adding 12 M hydro­chloric acid, which gave a white suspension. After the removal of the white powder of *S*-benzyl-*D*-penicillamine, the colorless filtrate was stood at room temperature for a month. A small amount of colorless platelet crystals of the title compound appeared on the wall of the glass vessel, one of which was used for single-crystal X-ray analysis.

### S3. Refinement

H atoms bound to C atoms were placed at calculated positions [C—H = 1.00 (CH), 0.99 (CH_2_), and 0.98 (CH_3_)] and refined as riding models with *U*_iso_(H) = 1.2*U*_eq_(C) for CH_2_ and CH, and *U*_iso_(H) = 1.5*U*_eq_(C) for CH_3_. All H atoms bound to N and O atoms were found in a difference Fourier map and their positions were refined with *U*_iso_(H) = 1.2*U*_eq_(N or O). Reflections of (-6 0 32), (-13 1 23), (8 0 18) and (-17 1 21) were removed to improve the data quality.

### Crystal data

**Table:**

C_19_H_23_NO_2_S·H_2_O	*F*(000) = 1488
*M**_r_* = 347.46	*D*_x_ = 1.223 Mg m^−^^3^
Monoclinic, *C*2	Mo *K*α radiation, λ = 0.71075 Å
*a* = 19.930 (2) Å	Cell parameters from 786 reflections
*b* = 6.2500 (7) Å	θ = 3.4–27.5°
*c* = 30.645 (4) Å	µ = 0.19 mm^−^^1^
β = 98.715 (7)°	*T* = 200 K
*V* = 3773.2 (8) Å^3^	Platelet, colorless
*Z* = 8	0.15 × 0.10 × 0.03 mm

### Data collection

**Table:**

Rigaku R-AXIS RAPID diffractometer	7694 independent reflections
Radiation source: fine-focus sealed tube	7006 reflections with *I* > 2σ(*I*)
Detector resolution: 10.00 pixels mm^-1^	*R*_int_ = 0.027
ω scans	θ_max_ = 27.5°, θ_min_ = 3.1°
Absorption correction: multi-scan (*ABSCOR*; Higashi, 1995)	*h* = −25→25
*T*_min_ = 0.778, *T*_max_ = 0.994	*k* = −8→8
15147 measured reflections	*l* = −39→39

### Refinement

**Table:**

Refinement on *F*^2^	Hydrogen site location: mixed
Least-squares matrix: full	H atoms treated by a mixture of independent and constrained refinement
*R*[*F*^2^ > 2σ(*F*^2^)] = 0.041	*w* = 1/[σ^2^(*F*_o_^2^) + (0.0482*P*)^2^ + 1.222*P*] where *P* = (*F*_o_^2^ + 2*F*_c_^2^)/3
*wR*(*F*^2^) = 0.093	(Δ/σ)_max_ < 0.001
*S* = 1.06	Δρ_max_ = 0.44 e Å^−^^3^
7694 reflections	Δρ_min_ = −0.25 e Å^−^^3^
461 parameters	Absolute structure: Flack *x* determined using 2566 quotients [(*I*^+^)-(*I*^-^)]/[(*I*^+^)+(*I*^-^)] (Parsons *et al.*, 2013)
1 restraint	Absolute structure parameter: 0.03 (2)

### Special details

**Table:**

Geometry. All e.s.d.'s (except the e.s.d. in the dihedral angle between two l.s. planes) are estimated using the full covariance matrix. The cell e.s.d.'s are taken into account individually in the estimation of e.s.d.'s in distances, angles and torsion angles; correlations between e.s.d.'s in cell parameters are only used when they are defined by crystal symmetry. An approximate (isotropic) treatment of cell e.s.d.'s is used for estimating e.s.d.'s involving l.s. planes.

### Fractional atomic coordinates and isotropic or equivalent isotropic displacement parameters (Å^2^)

**Table:**

	*x*	*y*	*z*	*U*_iso_*/*U*_eq_	
S1	0.54021 (3)	0.37960 (9)	0.07484 (2)	0.02754 (16)	
O1	0.53088 (10)	−0.0974 (3)	0.19789 (6)	0.0392 (5)	
O2	0.60252 (10)	−0.2717 (3)	0.16081 (6)	0.0349 (5)	
N1	0.58790 (11)	0.2829 (3)	0.17415 (7)	0.0240 (4)	
H1	0.5905 (13)	0.407 (5)	0.1604 (9)	0.029*	
H2	0.5508 (14)	0.309 (5)	0.1889 (9)	0.029*	
C1	0.52331 (12)	0.1335 (4)	0.10419 (8)	0.0244 (5)	
C2	0.58099 (12)	0.0945 (4)	0.14360 (8)	0.0234 (5)	
H3	0.6245	0.0792	0.1314	0.028*	
C3	0.57073 (12)	−0.1113 (4)	0.16993 (8)	0.0260 (5)	
C4	0.51755 (14)	−0.0596 (4)	0.07331 (9)	0.0347 (6)	
H4	0.5008	−0.1831	0.0882	0.052*	
H5	0.5623	−0.0930	0.0655	0.052*	
H6	0.4859	−0.0268	0.0464	0.052*	
C5	0.45430 (13)	0.1776 (5)	0.11894 (10)	0.0356 (6)	
H7	0.4435	0.0616	0.1382	0.053*	
H8	0.4191	0.1858	0.0930	0.053*	
H9	0.4563	0.3135	0.1350	0.053*	
C6	0.6145 (2)	0.3154 (6)	0.04975 (14)	0.0591 (10)	
H10	0.6489	0.2435	0.0716	0.071*	
H11	0.6019	0.2172	0.0245	0.071*	
C7	0.64320 (14)	0.5184 (5)	0.03420 (10)	0.0335 (6)	
C8	0.62917 (15)	0.5776 (6)	−0.00969 (10)	0.0458 (8)	
H12	0.6005	0.4913	−0.0302	0.055*	
C9	0.65753 (18)	0.7659 (6)	−0.02381 (12)	0.0578 (10)	
H13	0.6477	0.8079	−0.0539	0.069*	
C10	0.69916 (16)	0.8889 (6)	0.00542 (15)	0.0574 (10)	
H14	0.7189	1.0148	−0.0045	0.069*	
C11	0.71268 (18)	0.8333 (6)	0.04855 (15)	0.0601 (10)	
H15	0.7414	0.9205	0.0688	0.072*	
C12	0.68468 (17)	0.6499 (6)	0.06289 (11)	0.0501 (8)	
H16	0.6941	0.6127	0.0933	0.060*	
C13	0.64745 (13)	0.2701 (4)	0.21067 (8)	0.0318 (6)	
H17	0.6487	0.4014	0.2289	0.038*	
H18	0.6410	0.1468	0.2299	0.038*	
C14	0.71428 (14)	0.2462 (5)	0.19426 (9)	0.0308 (6)	
C15	0.74302 (14)	0.4186 (5)	0.17595 (9)	0.0387 (6)	
H19	0.7200	0.5520	0.1730	0.046*	
C16	0.80556 (15)	0.3974 (6)	0.16177 (12)	0.0529 (9)	
H20	0.8247	0.5154	0.1484	0.063*	
C17	0.84014 (16)	0.2054 (7)	0.16701 (12)	0.0557 (9)	
H21	0.8835	0.1928	0.1580	0.067*	
C18	0.81217 (16)	0.0333 (6)	0.18518 (12)	0.0545 (9)	
H22	0.8359	−0.0990	0.1884	0.065*	
C19	0.74940 (15)	0.0521 (5)	0.19883 (10)	0.0425 (7)	
H23	0.7300	−0.0677	0.2114	0.051*	
S2	0.46646 (3)	0.93252 (9)	0.42752 (2)	0.03026 (17)	
O3	0.50246 (10)	0.4534 (3)	0.30958 (6)	0.0380 (5)	
O4	0.42108 (11)	0.2829 (3)	0.33859 (7)	0.0391 (5)	
N2	0.43753 (12)	0.8345 (3)	0.32686 (7)	0.0254 (5)	
H24	0.4737 (15)	0.852 (5)	0.3142 (9)	0.030*	
H25	0.4328 (14)	0.955 (5)	0.3400 (9)	0.030*	
C20	0.49179 (13)	0.6890 (4)	0.40019 (8)	0.0276 (6)	
C21	0.44067 (13)	0.6488 (4)	0.35761 (8)	0.0249 (5)	
H26	0.3947	0.6325	0.3665	0.030*	
C22	0.45587 (13)	0.4405 (4)	0.33312 (8)	0.0275 (5)	
C23	0.49265 (17)	0.4945 (5)	0.43061 (9)	0.0402 (7)	
H27	0.5127	0.3722	0.4173	0.060*	
H28	0.4461	0.4594	0.4348	0.060*	
H29	0.5197	0.5273	0.4593	0.060*	
C24	0.56361 (14)	0.7409 (5)	0.39158 (10)	0.0398 (7)	
H30	0.5930	0.7668	0.4197	0.060*	
H31	0.5625	0.8690	0.3730	0.060*	
H32	0.5814	0.6201	0.3764	0.060*	
C25	0.3886 (2)	0.8563 (6)	0.44760 (15)	0.0630 (11)	
H33	0.3596	0.7698	0.4251	0.076*	
H34	0.3991	0.7703	0.4749	0.076*	
C26	0.35257 (14)	1.0599 (5)	0.45678 (10)	0.0365 (7)	
C27	0.3042 (2)	1.1515 (10)	0.42613 (13)	0.0805 (15)	
H35	0.2931	1.0879	0.3978	0.097*	
C28	0.2713 (3)	1.3345 (13)	0.4359 (3)	0.124 (3)	
H36	0.2370	1.3944	0.4145	0.149*	
C29	0.2872 (3)	1.4297 (8)	0.4754 (3)	0.110 (3)	
H37	0.2646	1.5575	0.4817	0.133*	
C30	0.3356 (2)	1.3424 (7)	0.50638 (17)	0.0781 (15)	
H38	0.3468	1.4092	0.5344	0.094*	
C31	0.36859 (16)	1.1567 (6)	0.49720 (10)	0.0447 (8)	
H39	0.4023	1.0961	0.5189	0.054*	
C32	0.38062 (14)	0.8229 (5)	0.28808 (9)	0.0334 (6)	
H40	0.3883	0.9333	0.2662	0.040*	
H41	0.3819	0.6813	0.2737	0.040*	
C33	0.31154 (14)	0.8556 (5)	0.30101 (9)	0.0339 (6)	
C34	0.28147 (16)	1.0576 (6)	0.29587 (10)	0.0444 (8)	
H42	0.3056	1.1741	0.2857	0.053*	
C35	0.21634 (18)	1.0884 (7)	0.30561 (12)	0.0605 (11)	
H43	0.1958	1.2257	0.3021	0.073*	
C36	0.18146 (16)	0.9183 (8)	0.32053 (11)	0.0605 (11)	
H44	0.1369	0.9394	0.3271	0.073*	
C37	0.21099 (17)	0.7187 (7)	0.32586 (11)	0.0544 (9)	
H45	0.1869	0.6031	0.3363	0.065*	
C38	0.27558 (15)	0.6868 (6)	0.31597 (10)	0.0413 (7)	
H46	0.2956	0.5487	0.3194	0.050*	
O5	0.49439 (11)	0.4819 (3)	0.21838 (8)	0.0391 (5)	
H47	0.4939 (16)	0.483 (6)	0.2448 (11)	0.047*	
H48	0.5069 (17)	0.601 (6)	0.2145 (11)	0.047*	
O6	0.54027 (12)	0.0182 (4)	0.28774 (8)	0.0425 (5)	
H49	0.5289 (18)	0.141 (6)	0.2907 (11)	0.051*	
H50	0.5348 (17)	0.001 (6)	0.2613 (12)	0.051*	

### Atomic displacement parameters (Å^2^)

**Table:**

	*U*^11^	*U*^22^	*U*^33^	*U*^12^	*U*^13^	*U*^23^
S1	0.0323 (3)	0.0171 (3)	0.0342 (3)	0.0018 (2)	0.0084 (3)	0.0037 (3)
O1	0.0554 (12)	0.0240 (10)	0.0426 (11)	−0.0015 (9)	0.0219 (10)	0.0062 (9)
O2	0.0442 (11)	0.0158 (9)	0.0455 (11)	0.0028 (8)	0.0091 (9)	0.0017 (8)
N1	0.0281 (11)	0.0137 (10)	0.0321 (12)	−0.0022 (8)	0.0101 (10)	−0.0005 (8)
C1	0.0250 (12)	0.0157 (12)	0.0327 (13)	−0.0031 (9)	0.0048 (11)	0.0039 (10)
C2	0.0238 (12)	0.0150 (11)	0.0327 (13)	−0.0013 (9)	0.0082 (11)	−0.0002 (10)
C3	0.0311 (12)	0.0175 (12)	0.0284 (12)	−0.0029 (10)	0.0010 (11)	−0.0017 (10)
C4	0.0395 (14)	0.0210 (13)	0.0411 (15)	−0.0051 (12)	−0.0018 (12)	−0.0023 (12)
C5	0.0238 (13)	0.0365 (16)	0.0477 (16)	−0.0011 (11)	0.0088 (12)	0.0100 (13)
C6	0.071 (2)	0.0302 (17)	0.089 (3)	0.0130 (16)	0.053 (2)	0.0130 (17)
C7	0.0304 (14)	0.0277 (14)	0.0463 (16)	0.0041 (11)	0.0179 (13)	0.0057 (12)
C8	0.0330 (15)	0.056 (2)	0.0477 (18)	−0.0005 (14)	0.0025 (14)	0.0030 (16)
C9	0.050 (2)	0.069 (3)	0.057 (2)	0.0199 (19)	0.0183 (18)	0.033 (2)
C10	0.0424 (17)	0.0333 (18)	0.106 (3)	0.0004 (15)	0.040 (2)	0.008 (2)
C11	0.0464 (19)	0.052 (2)	0.086 (3)	−0.0097 (16)	0.022 (2)	−0.025 (2)
C12	0.0473 (19)	0.062 (2)	0.0436 (17)	0.0082 (17)	0.0142 (15)	−0.0038 (16)
C13	0.0409 (15)	0.0250 (14)	0.0288 (13)	−0.0049 (12)	0.0036 (12)	−0.0022 (11)
C14	0.0304 (14)	0.0285 (15)	0.0307 (13)	−0.0023 (11)	−0.0043 (11)	−0.0033 (11)
C15	0.0338 (13)	0.0333 (16)	0.0486 (16)	0.0028 (13)	0.0049 (12)	0.0040 (14)
C16	0.0364 (15)	0.057 (2)	0.067 (2)	−0.0038 (16)	0.0131 (15)	0.0060 (19)
C17	0.0285 (15)	0.069 (3)	0.069 (2)	0.0056 (16)	0.0063 (16)	−0.006 (2)
C18	0.0399 (17)	0.045 (2)	0.075 (2)	0.0126 (15)	−0.0035 (17)	−0.0053 (18)
C19	0.0408 (16)	0.0302 (16)	0.0532 (18)	−0.0008 (13)	−0.0035 (14)	−0.0016 (13)
S2	0.0398 (4)	0.0192 (4)	0.0337 (3)	−0.0036 (3)	0.0115 (3)	−0.0060 (3)
O3	0.0476 (11)	0.0289 (11)	0.0412 (10)	0.0018 (9)	0.0189 (9)	−0.0071 (9)
O4	0.0501 (12)	0.0178 (10)	0.0506 (12)	−0.0071 (9)	0.0119 (10)	−0.0058 (9)
N2	0.0319 (11)	0.0157 (10)	0.0300 (12)	−0.0018 (9)	0.0093 (10)	−0.0017 (9)
C20	0.0355 (14)	0.0175 (12)	0.0303 (13)	0.0024 (10)	0.0061 (12)	−0.0050 (10)
C21	0.0302 (13)	0.0159 (12)	0.0305 (13)	−0.0019 (10)	0.0108 (11)	−0.0024 (10)
C22	0.0346 (13)	0.0183 (12)	0.0291 (12)	0.0014 (11)	0.0031 (11)	−0.0005 (11)
C23	0.0590 (19)	0.0256 (15)	0.0337 (15)	0.0034 (13)	−0.0004 (14)	0.0007 (12)
C24	0.0330 (15)	0.0426 (18)	0.0437 (16)	−0.0013 (13)	0.0052 (13)	−0.0122 (14)
C25	0.071 (2)	0.039 (2)	0.091 (3)	−0.0139 (17)	0.054 (2)	−0.0174 (19)
C26	0.0333 (15)	0.0376 (17)	0.0424 (16)	−0.0056 (13)	0.0177 (13)	−0.0084 (13)
C27	0.050 (2)	0.143 (5)	0.047 (2)	−0.001 (3)	0.0041 (19)	0.017 (3)
C28	0.061 (3)	0.149 (7)	0.169 (6)	0.050 (4)	0.038 (4)	0.107 (5)
C29	0.070 (3)	0.042 (3)	0.243 (8)	0.014 (2)	0.096 (5)	0.020 (4)
C30	0.072 (3)	0.065 (3)	0.111 (3)	−0.033 (2)	0.057 (3)	−0.052 (3)
C31	0.0386 (16)	0.052 (2)	0.0454 (17)	−0.0048 (14)	0.0122 (14)	−0.0092 (15)
C32	0.0427 (15)	0.0287 (14)	0.0281 (13)	0.0011 (12)	0.0029 (12)	−0.0006 (11)
C33	0.0357 (14)	0.0324 (15)	0.0311 (14)	−0.0018 (12)	−0.0032 (12)	−0.0081 (11)
C34	0.0443 (17)	0.0398 (19)	0.0446 (17)	0.0038 (14)	−0.0076 (14)	−0.0110 (14)
C35	0.049 (2)	0.060 (2)	0.065 (2)	0.0200 (18)	−0.0163 (18)	−0.026 (2)
C36	0.0316 (15)	0.093 (3)	0.055 (2)	0.006 (2)	−0.0012 (15)	−0.033 (2)
C37	0.0389 (18)	0.074 (3)	0.0501 (19)	−0.0087 (18)	0.0062 (16)	−0.0110 (19)
C38	0.0381 (16)	0.0424 (17)	0.0420 (16)	−0.0028 (13)	0.0021 (14)	−0.0072 (14)
O5	0.0536 (13)	0.0256 (11)	0.0425 (11)	0.0005 (9)	0.0214 (10)	0.0005 (9)
O6	0.0581 (14)	0.0281 (11)	0.0461 (12)	−0.0041 (10)	0.0236 (11)	−0.0020 (10)

### Geometric parameters (Å, º)

**Table:**

S1—C6	1.814 (3)	O3—C22	1.262 (3)
S1—C1	1.839 (2)	O4—C22	1.230 (3)
O1—C3	1.256 (3)	N2—C21	1.490 (3)
O2—C3	1.240 (3)	N2—C32	1.515 (4)
N1—C2	1.497 (3)	N2—H24	0.88 (3)
N1—C13	1.505 (3)	N2—H25	0.87 (3)
N1—H1	0.89 (3)	C20—C24	1.529 (4)
N1—H2	0.94 (3)	C20—C23	1.531 (4)
C1—C4	1.527 (4)	C20—C21	1.549 (4)
C1—C5	1.536 (3)	C21—C22	1.556 (3)
C1—C2	1.555 (3)	C21—H26	1.0000
C2—C3	1.549 (3)	C23—H27	0.9800
C2—H3	1.0000	C23—H28	0.9800
C4—H4	0.9800	C23—H29	0.9800
C4—H5	0.9800	C24—H30	0.9800
C4—H6	0.9800	C24—H31	0.9800
C5—H7	0.9800	C24—H32	0.9800
C5—H8	0.9800	C25—C26	1.508 (5)
C5—H9	0.9800	C25—H33	0.9900
C6—C7	1.498 (4)	C25—H34	0.9900
C6—H10	0.9900	C26—C27	1.366 (5)
C6—H11	0.9900	C26—C31	1.372 (4)
C7—C8	1.382 (4)	C27—C28	1.373 (9)
C7—C12	1.383 (5)	C27—H35	0.9500
C8—C9	1.402 (5)	C28—C29	1.343 (9)
C8—H12	0.9500	C28—H36	0.9500
C9—C10	1.363 (5)	C29—C30	1.362 (8)
C9—H13	0.9500	C29—H37	0.9500
C10—C11	1.354 (6)	C30—C31	1.384 (6)
C10—H14	0.9500	C30—H38	0.9500
C11—C12	1.376 (5)	C31—H39	0.9500
C11—H15	0.9500	C32—C33	1.503 (4)
C12—H16	0.9500	C32—H40	0.9900
C13—C14	1.501 (4)	C32—H41	0.9900
C13—H17	0.9900	C33—C38	1.392 (4)
C13—H18	0.9900	C33—C34	1.396 (4)
C14—C15	1.379 (4)	C34—C35	1.389 (5)
C14—C19	1.397 (4)	C34—H42	0.9500
C15—C16	1.387 (4)	C35—C36	1.385 (6)
C15—H19	0.9500	C35—H43	0.9500
C16—C17	1.381 (5)	C36—C37	1.378 (6)
C16—H20	0.9500	C36—H44	0.9500
C17—C18	1.368 (5)	C37—C38	1.381 (4)
C17—H21	0.9500	C37—H45	0.9500
C18—C19	1.383 (4)	C38—H46	0.9500
C18—H22	0.9500	O5—H47	0.81 (3)
C19—H23	0.9500	O5—H48	0.80 (4)
S2—C25	1.817 (3)	O6—H49	0.81 (4)
S2—C20	1.844 (3)	O6—H50	0.81 (3)
			
C6—S1—C1	104.14 (14)	C21—N2—C32	114.4 (2)
C2—N1—C13	114.3 (2)	C21—N2—H24	114.7 (19)
C2—N1—H1	113.4 (18)	C32—N2—H24	103.1 (18)
C13—N1—H1	107.6 (18)	C21—N2—H25	112.5 (19)
C2—N1—H2	115.6 (18)	C32—N2—H25	106.5 (19)
C13—N1—H2	104.0 (17)	H24—N2—H25	105 (3)
H1—N1—H2	101 (2)	C24—C20—C23	110.4 (2)
C4—C1—C5	109.7 (2)	C24—C20—C21	113.7 (2)
C4—C1—C2	109.6 (2)	C23—C20—C21	109.2 (2)
C5—C1—C2	112.9 (2)	C24—C20—S2	103.38 (18)
C4—C1—S1	111.12 (17)	C23—C20—S2	110.94 (17)
C5—C1—S1	103.44 (17)	C21—C20—S2	109.02 (17)
C2—C1—S1	110.01 (16)	N2—C21—C20	111.4 (2)
N1—C2—C3	109.52 (19)	N2—C21—C22	109.84 (18)
N1—C2—C1	110.5 (2)	C20—C21—C22	113.0 (2)
C3—C2—C1	113.22 (19)	N2—C21—H26	107.4
N1—C2—H3	107.8	C20—C21—H26	107.4
C3—C2—H3	107.8	C22—C21—H26	107.4
C1—C2—H3	107.8	O4—C22—O3	127.4 (2)
O2—C3—O1	127.4 (2)	O4—C22—C21	116.7 (2)
O2—C3—C2	116.2 (2)	O3—C22—C21	115.9 (2)
O1—C3—C2	116.4 (2)	C20—C23—H27	109.5
C1—C4—H4	109.5	C20—C23—H28	109.5
C1—C4—H5	109.5	H27—C23—H28	109.5
H4—C4—H5	109.5	C20—C23—H29	109.5
C1—C4—H6	109.5	H27—C23—H29	109.5
H4—C4—H6	109.5	H28—C23—H29	109.5
H5—C4—H6	109.5	C20—C24—H30	109.5
C1—C5—H7	109.5	C20—C24—H31	109.5
C1—C5—H8	109.5	H30—C24—H31	109.5
H7—C5—H8	109.5	C20—C24—H32	109.5
C1—C5—H9	109.5	H30—C24—H32	109.5
H7—C5—H9	109.5	H31—C24—H32	109.5
H8—C5—H9	109.5	C26—C25—S2	107.3 (2)
C7—C6—S1	108.8 (2)	C26—C25—H33	110.3
C7—C6—H10	109.9	S2—C25—H33	110.3
S1—C6—H10	109.9	C26—C25—H34	110.3
C7—C6—H11	109.9	S2—C25—H34	110.3
S1—C6—H11	109.9	H33—C25—H34	108.5
H10—C6—H11	108.3	C27—C26—C31	118.7 (4)
C8—C7—C12	118.2 (3)	C27—C26—C25	122.1 (4)
C8—C7—C6	120.4 (3)	C31—C26—C25	119.2 (3)
C12—C7—C6	121.4 (3)	C26—C27—C28	120.6 (5)
C7—C8—C9	119.6 (3)	C26—C27—H35	119.7
C7—C8—H12	120.2	C28—C27—H35	119.7
C9—C8—H12	120.2	C29—C28—C27	120.8 (5)
C10—C9—C8	120.3 (3)	C29—C28—H36	119.6
C10—C9—H13	119.9	C27—C28—H36	119.6
C8—C9—H13	119.9	C28—C29—C30	119.7 (5)
C11—C10—C9	120.5 (3)	C28—C29—H37	120.2
C11—C10—H14	119.7	C30—C29—H37	120.2
C9—C10—H14	119.7	C29—C30—C31	120.2 (4)
C10—C11—C12	119.7 (4)	C29—C30—H38	119.9
C10—C11—H15	120.1	C31—C30—H38	119.9
C12—C11—H15	120.1	C26—C31—C30	120.1 (4)
C11—C12—C7	121.6 (3)	C26—C31—H39	120.0
C11—C12—H16	119.2	C30—C31—H39	120.0
C7—C12—H16	119.2	C33—C32—N2	113.2 (2)
C14—C13—N1	113.3 (2)	C33—C32—H40	108.9
C14—C13—H17	108.9	N2—C32—H40	108.9
N1—C13—H17	108.9	C33—C32—H41	108.9
C14—C13—H18	108.9	N2—C32—H41	108.9
N1—C13—H18	108.9	H40—C32—H41	107.8
H17—C13—H18	107.7	C38—C33—C34	119.3 (3)
C15—C14—C19	119.1 (3)	C38—C33—C32	121.4 (3)
C15—C14—C13	120.2 (3)	C34—C33—C32	119.2 (3)
C19—C14—C13	120.6 (3)	C35—C34—C33	120.0 (3)
C14—C15—C16	120.1 (3)	C35—C34—H42	120.0
C14—C15—H19	119.9	C33—C34—H42	120.0
C16—C15—H19	119.9	C36—C35—C34	119.8 (3)
C17—C16—C15	120.2 (3)	C36—C35—H43	120.1
C17—C16—H20	119.9	C34—C35—H43	120.1
C15—C16—H20	119.9	C37—C36—C35	120.5 (3)
C18—C17—C16	120.3 (3)	C37—C36—H44	119.8
C18—C17—H21	119.9	C35—C36—H44	119.8
C16—C17—H21	119.9	C36—C37—C38	120.0 (4)
C17—C18—C19	119.9 (3)	C36—C37—H45	120.0
C17—C18—H22	120.0	C38—C37—H45	120.0
C19—C18—H22	120.0	C37—C38—C33	120.4 (3)
C18—C19—C14	120.4 (3)	C37—C38—H46	119.8
C18—C19—H23	119.8	C33—C38—H46	119.8
C14—C19—H23	119.8	H47—O5—H48	101 (4)
C25—S2—C20	104.13 (14)	H49—O6—H50	104 (4)
			
C6—S1—C1—C4	−53.7 (2)	C25—S2—C20—C24	−169.3 (2)
C6—S1—C1—C5	−171.4 (2)	C25—S2—C20—C23	−51.0 (3)
C6—S1—C1—C2	67.8 (2)	C25—S2—C20—C21	69.4 (2)
C13—N1—C2—C3	60.9 (3)	C32—N2—C21—C20	−170.4 (2)
C13—N1—C2—C1	−173.69 (19)	C32—N2—C21—C22	63.5 (3)
C4—C1—C2—N1	179.6 (2)	C24—C20—C21—N2	−56.5 (3)
C5—C1—C2—N1	−57.9 (3)	C23—C20—C21—N2	179.7 (2)
S1—C1—C2—N1	57.1 (2)	S2—C20—C21—N2	58.3 (2)
C4—C1—C2—C3	−57.2 (3)	C24—C20—C21—C22	67.8 (3)
C5—C1—C2—C3	65.4 (3)	C23—C20—C21—C22	−56.1 (3)
S1—C1—C2—C3	−179.64 (16)	S2—C20—C21—C22	−177.47 (17)
N1—C2—C3—O2	−137.9 (2)	N2—C21—C22—O4	−134.3 (2)
C1—C2—C3—O2	98.4 (3)	C20—C21—C22—O4	100.6 (3)
N1—C2—C3—O1	42.6 (3)	N2—C21—C22—O3	45.3 (3)
C1—C2—C3—O1	−81.2 (3)	C20—C21—C22—O3	−79.8 (3)
C1—S1—C6—C7	−166.4 (3)	C20—S2—C25—C26	−161.7 (2)
S1—C6—C7—C8	−99.3 (3)	S2—C25—C26—C27	93.5 (4)
S1—C6—C7—C12	81.4 (4)	S2—C25—C26—C31	−86.6 (3)
C12—C7—C8—C9	0.6 (4)	C31—C26—C27—C28	−1.2 (6)
C6—C7—C8—C9	−178.7 (3)	C25—C26—C27—C28	178.6 (4)
C7—C8—C9—C10	0.6 (5)	C26—C27—C28—C29	1.5 (8)
C8—C9—C10—C11	−1.2 (5)	C27—C28—C29—C30	−0.9 (9)
C9—C10—C11—C12	0.6 (5)	C28—C29—C30—C31	0.2 (7)
C10—C11—C12—C7	0.6 (5)	C27—C26—C31—C30	0.4 (5)
C8—C7—C12—C11	−1.2 (5)	C25—C26—C31—C30	−179.4 (3)
C6—C7—C12—C11	178.1 (3)	C29—C30—C31—C26	0.1 (5)
C2—N1—C13—C14	57.6 (3)	C21—N2—C32—C33	71.1 (3)
N1—C13—C14—C15	74.4 (3)	N2—C32—C33—C38	−85.2 (3)
N1—C13—C14—C19	−108.1 (3)	N2—C32—C33—C34	97.9 (3)
C19—C14—C15—C16	0.9 (4)	C38—C33—C34—C35	−0.1 (4)
C13—C14—C15—C16	178.5 (3)	C32—C33—C34—C35	176.9 (3)
C14—C15—C16—C17	−1.7 (5)	C33—C34—C35—C36	0.2 (5)
C15—C16—C17—C18	1.7 (5)	C34—C35—C36—C37	0.1 (5)
C16—C17—C18—C19	−0.7 (5)	C35—C36—C37—C38	−0.5 (5)
C17—C18—C19—C14	−0.1 (5)	C36—C37—C38—C33	0.6 (5)
C15—C14—C19—C18	0.0 (4)	C34—C33—C38—C37	−0.3 (4)
C13—C14—C19—C18	−177.5 (3)	C32—C33—C38—C37	−177.2 (3)

### Hydrogen-bond geometry (Å, º)

**Table:**

*D*—H···*A*	*D*—H	H···*A*	*D*···*A*	*D*—H···*A*
N1—H1···S1	0.89 (3)	2.67 (3)	3.107 (2)	112 (2)
N1—H1···O2^i^	0.89 (3)	2.02 (3)	2.835 (3)	152 (2)
N1—H2···O5	0.94 (3)	1.89 (3)	2.761 (3)	154 (3)
N2—H24···O6^i^	0.88 (3)	1.96 (3)	2.774 (3)	155 (3)
N2—H25···S2	0.87 (3)	2.67 (3)	3.112 (2)	113 (2)
N2—H25···O4^i^	0.87 (3)	2.06 (3)	2.851 (3)	151 (3)
O5—H47···O3	0.81 (3)	1.97 (3)	2.781 (3)	172 (4)
O5—H48···O1^i^	0.80 (4)	2.03 (4)	2.824 (3)	173 (3)
O6—H49···O3	0.81 (4)	2.13 (4)	2.927 (3)	171 (3)
O6—H50···O1	0.81 (3)	2.03 (4)	2.825 (3)	169 (4)

Symmetry code: (i) *x*, *y*+1, *z*.
