# Supplementary figures and images for: Crystal structure of S,N-dibenzyl-d-penicillamine monohydrate
Source: Acta Crystallogr Sect E Struct Rep Online. 2014 Oct 29;70(Pt 11):o1209. doi: 10.1107/S1600536814023459 (PMC4257258; doi:10.1107/S1600536814023459)

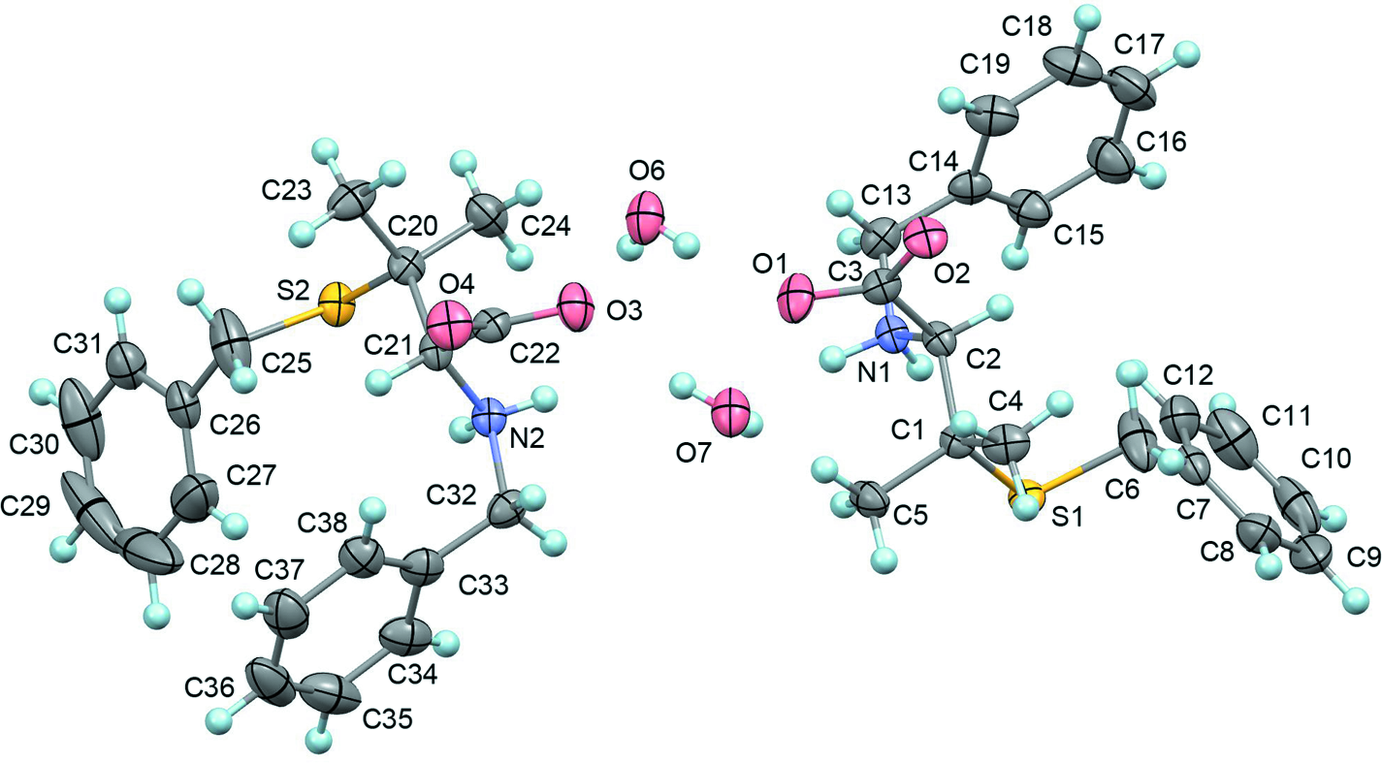

Supplement: Supplementary file 3 [file e-70-o1209-fig1.tif]

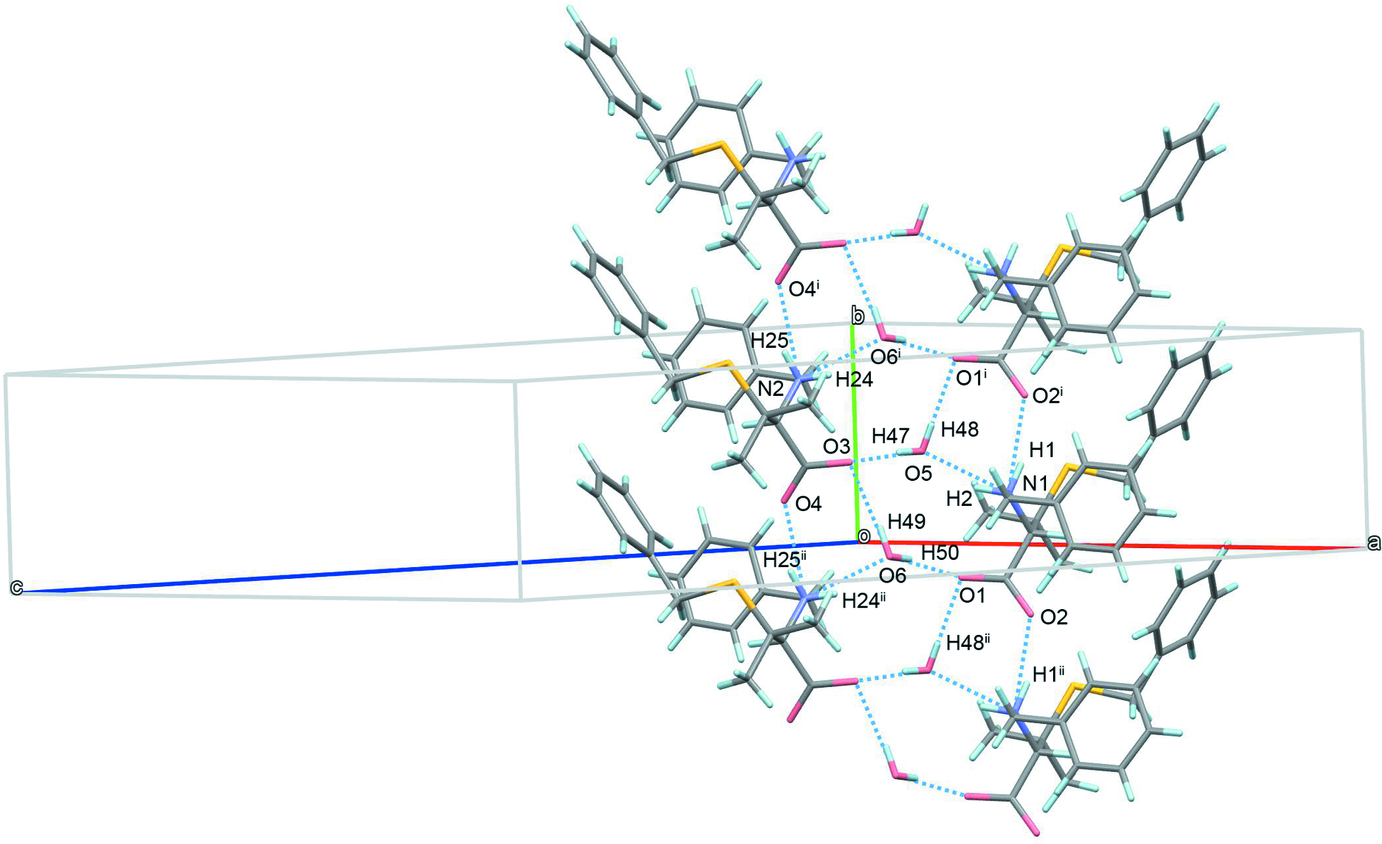

Supplement: Supplementary file 4 [file e-70-o1209-fig2.tif]
